# Editorial: ARDS: Reaching for the Horizon

**DOI:** 10.3389/fped.2017.00100

**Published:** 2017-05-15

**Authors:** John K. McGuire, Andreas Schwingshackl, Kanwaljeet J. S. Anand

**Affiliations:** ^1^Pediatric Critical Care Medicine, University of Washington, Seattle, WA, USA; ^2^Pediatric Critical Care Medicine, University of California Los Angeles, Los Angeles, CA, USA; ^3^Department of Pediatrics, Stanford University, Stanford, CA, USA

**Keywords:** pediatric acute respiratory distress syndrome, acute lung injury, pathophysiology, treatment, fluid management

**Editorial on the Research Topic**

**ARDS: Reaching for the Horizon**

The acute respiratory distress syndrome (ARDS) represents a syndrome of severe diffuse, hypoxemic, inflammatory lung disease caused by a broad range of pulmonary and non-pulmonary processes. Since Ashbaugh and Petty first ascribed the term ARDS (initially adult respiratory distress syndrome) in their landmark 1967 report (1), five decades of study reinforce early observations that ARDS is not limited to adults and it is different in children and newborns. However, most of the approaches to diagnosis and treatment of critically ill children with ARDS have been extrapolated from studies in adults. A major step toward the goal of specific pediatric ARDS (PARDS) recommendations was the convening of the Pediatric Acute Lung Injury Consensus Conference (PALICC) to use best available data and expert consensus to create guidelines for PARDS definitions, treatment, and research priorities (2). On this foundation, we present a special collection of 13 state-of-the-art reviews that address key areas in PARDS biology and pathophysiology, recognition and diagnosis, and treatment. Of particular interest is the consideration of unique pediatric situations including bronchopulmonary dysplasia and severe pertussis and some of the more controversial aspects of PARDS management such as steroid therapy and fluid management.

## PARDS Etiologies and Epidemiology

Every pediatric care provider is intimately familiar with the impact of respiratory infections on children, which remains among the leading causes of hospitalization in children (3). Nye et al. provide an in-depth consideration of the role of respiratory syncytial virus (RSV) and influenza, as well as less frequent viruses as causes of ARDS in children. Common among the respiratory viruses are the targeting of the epithelium, intense pulmonary inflammatory responses, and similar risk factors for severe infection leading to ARDS, including younger age, underlying chronic lung disease, and an immunocompromised condition. As newer antiviral agents become available, rapid methods for diagnosis of specific viruses will be increasingly valuable to implement specific treatment(s) as early as possible. The immunocompromised child bears special mention as more aggressive regimens for autoimmune disease, cancer, and posttransplant management increase the prevalence of children with impaired immunity at risk for severe disease from common viruses. A reemerging cause of severe lung disease, especially in very young children, is *Bordetella pertussis*. Severe pertussis has not been characterized as ARDS *per se*, but the manifestations seen in young infants with bilateral pneumonia, hypoxemia, pulmonary hypertension, and a potentially fulminant course merit inclusion of Zlamy’s fantastic overview of pertussis biology and clinical features in children as well as strategies for prevention.

Despite the marked increase in numbers of children living with chronic lung disease, the application of the PALICC PARDS diagnosis guidelines is challenging in those with underlying perinatal lung disease (4). Bhandari et al. present a very interesting consideration of bronchopulmonary dysplasia (BPD), the most common chronic respiratory disease in premature infants, as a substrate for ARDS. They highlight the unique pathophysiology and consequences of lung injury in the developing lung and the similarities in biology between BPD and ARDS, including inflammatory cytokines, matrix metalloproteinases, and angiopoietins. Current data do not associate BPD as a risk factor for PARDS or as a determinant of outcome; however, the categorization and definition of ARDS in children with underlying conditions such as BPD may be of critical importance in guiding therapy (Bhandari et al.). Indeed, bronchodilator and steroid therapy are mainstays of treating BPD exacerbations yet remain of uncertain value in PARDS management (5).

## PARDS Recognition/Diagnosis

Since much of the care of ARDS is currently supportive, why do definitions matter so much? We argue that the diagnosis of ARDS heightens the clinician’s level of concern and puts the care in the context of significant risks. Although clinical criteria aid in making a diagnosis at the bedside, advances in biomarker identification and molecular phenotyping have the potential to identify those patients at high risk for poor outcomes and perhaps inform the use of targeted therapies for specific groups of patients. The great shortcoming of studies of targeted therapies in ARDS is that enrollment criteria based on a handful of clinical features predictably does not match individual patients to treatments that might target their unique pathology. Addressing this need for patient-level understanding, Orwoll and Sapru provide a comprehensive review of the current state of PARDS biomarkers in the context of lung injury pathophysiology. As an example of the promise that biomarkers may serve as surrogate outcomes for PARDS, Kimura et al. present results of their pilot study testing the potential use of biomarkers as correlates of clinical response in a small trial of low-dose methylprednisolone therapy for children with ARDS. They observed that methylprednisolone treatment was associated with lower neutrophil activation (MMP-8), decreased endothelial injury (sICAM-1), and enhanced epithelial recovery (sRAGE). Results such as these put clinical outcomes in the individual patient pathophysiological context and provide important preliminary data for planning larger multicentered studies with surrogate outcomes other than mortality, which is challenging to use in PARDS.

## PARDS Treatment

Treatment for PARDS remains largely limited to “supportive care.” However, what that means and how to best provide it is not at all clear. Among the strategies considered are those for which there is strong agreement of the value, such as the need to maintain patient comfort and cooperation with mechanical ventilation and to provide adequate nutrition. Vet et al. provide an overview of the medication choices and goals of sedation in PARDS, including the importance of non-pharmacologic strategies and recognizing the risks of delirium. In the future, it is likely that the focus will shift from keeping children sedated to keeping them “comfortable,” which for some children may mean awake with minimal sedation. Provision of nutrition in critically ill children is a cornerstone of PICU care. Wilson and Typpo provide an in-depth overview of the available data on impact of nutrition in ARDS (mostly from adult studies) and present recommendations for several key topics such as enteral vs. parenteral nutrition, what to the feed and when, and the potential for dietary immunomodulation. Another area in which the importance is clear but what to do is not clear is fluid management in PARDS. Ingelse et al.’s systematic review reinforces the growing consensus that fluid overload in PARDS is associated with worse outcomes. They also address the critical question of how to avoid fluid overload (restriction vs. diuretics vs. renal replacement therapy) and make a strong case for the necessity for age-related fluid management protocols.

Although for fluid management, a consensus of “enough but not too much” may be emerging, there is certainly little consensus on the use of glucocorticoid therapy in PARDS. Addressing the controversy head on, we commissioned a PRO-CON debate from some of the leading experts in the field (Hartmann and Hough; Schwingshackl and Meduri). Taken together, the two manuscripts and a third by Meduri et al. considering the impact of steroids on ICU-acquired weakness present a stimulating discussion of the rationale, benefits, and potential risks of steroid use in PARDS and lead us to a joint conclusion that more study is clearly needed. Although one of the proposed advantages of glucocorticoids is their low cost, another controversial therapy with a high cost is inhaled nitric oxide (iNO). Multiple studies of iNO in adult ARDS have not shown benefit on mortality or liberation from mechanical ventilation. However, a recent study by Bronicki et al. showed a decreased duration of mechanical ventilation and an increased rate of ECMO-free survival in PARDS patients who were randomized to receiving iNO (6). As a follow-up, Hunt et al. put these results in the context of how PARDS pathophysiology differs from that of adults and also address the PALICC recommendation against the routine use of iNO in PARDS in the context of more targeted use through PARDS patient subclass stratification.

## PARDS Long-Term Outcomes and Future Directions

Over the past few decades, PARDS mortality has decreased over threefold to less than 20% (7, 8). However, among survivors, the impact of long-term morbidity in children after critical illness is being increasingly recognized (9). Pertaining to PARDS, Im et al. provide a broad overview of the biological consequences of severe lung injury and of the ARDS treatments on outcomes such as pulmonary fibrosis. They highlight the potential for significant long-term improvement and make the case for understanding how to enhance lung repair, promote resolution of inflammation, and inhibit fibrosis.

Because of the changing technology and a relative paucity of evidence, this collection does not include any reviews on ventilator management, including the use of high-frequency oscillatory ventilation (10) or other lung recruitment maneuvers (11), or retrospective data analyses for identifying prognostic variables of clinical outcomes in PARDS (8). Taken together, these state-of-the-art reviews reflect on the progress made since the initial ARDS report nearly five decades ago and engender optimism as we “Reach for the Horizon” of increased understanding of PARDS diagnosis, biomarkers, treatment, and improved long-term outcomes.

## Author Contributions

JM, AS, and KA contributed to the development of this research topic and the accompanying editorial, as well as wrote and edited the editorial manuscript.

## Conflict of Interest Statement

The authors declare that the research was conducted in the absence of any commercial or financial relationships that could be construed as a potential conflict of interest.
